# The long-term cost-effectiveness of once-weekly semaglutide 1 mg vs. dulaglutide 3 mg and 4.5 mg in the UK

**DOI:** 10.1007/s10198-022-01514-1

**Published:** 2022-09-17

**Authors:** Adie Viljoen, Barrie Chubb, Samuel J. P. Malkin, Sasha Berry, Barnaby Hunt, Stephen C. Bain

**Affiliations:** 1 Borthwick Diabetes Research Centre, Lister Hospital (East and North Hertfordshire NHS Trust), Stevenage, UK; 2 Novo Nordisk Ltd, Gatwick, UK; 3 Ossian Health Economics and Communications GmbH, Bäumleingasse 20, 4051 Basel, Switzerland; 4 Swansea University Medical School, Swansea, UK

**Keywords:** Cost, Cost-effectiveness, Semaglutide, Dulaglutide, UK, D61, I19

## Abstract

**Aims:**

Once-weekly semaglutide and dulaglutide represent two highly efficacious treatment options for type 2 diabetes. A recent indirect treatment comparison (ITC) has associated semaglutide 1 mg with similar and greater improvements in glycated haemoglobin (HbA1c) and body weight, respectively, vs. dulaglutide 3 mg and 4.5 mg. The present study aimed to evaluate the long-term cost-effectiveness of semaglutide 1 mg vs. dulaglutide 3 mg and 4.5 mg in the UK.

**Materials and methods:**

The IQVIA CORE Diabetes Model (v9.0) was used to project outcomes over patients’ lifetimes. Baseline cohort characteristics were sourced from SUSTAIN 7, with changes in HbA1c and body mass index applied as per the ITC. Modelled patients received semaglutide or dulaglutide for 3 years, after which treatment was intensified to basal insulin. Costs (expressed in 2020 pounds sterling [GBP]) were accounted from a healthcare payer perspective.

**Results:**

Semaglutide 1 mg was associated with improvements in quality-adjusted life expectancy of 0.05 and 0.04 quality-adjusted life years (QALYs) vs. dulaglutide 3 mg and 4.5 mg, respectively, due to a reduced incidence of diabetes-related complications with semaglutide. Direct costs were estimated to be GBP 76 lower and GBP 8 higher in the comparisons with dulaglutide 3 mg and 4.5 mg, respectively. Overall outcomes were similar, but favoured semaglutide, and based on modelled mean outcomes it was considered dominant vs. dulaglutide 3 mg and associated with an incremental cost-effectiveness ratio of GBP 228 per QALY gained vs. dulaglutide 4.5 mg.

**Conclusions:**

Semaglutide 1 mg represents a cost-effective treatment vs. dulaglutide 3 mg and 4.5 mg for type 2 diabetes from a healthcare payer perspective in the UK.

**Supplementary Information:**

The online version contains supplementary material available at 10.1007/s10198-022-01514-1.

## Introduction

Reducing the incidence of long-term complications attributable to type 2 diabetes is vital to minimising the high and increasing expenditure associated with the disease. Total diabetes-related expenditure in the UK was estimated to be more than GBP 17 billion in 2021, with projections indicating that the prevalence of diabetes is expected to increase from 6.3% in 2021 to 7.5% by 2045 [1]. Improving glycaemic control remains a key target of care based on landmark studies, including the United Kingdom Prospective Diabetes Study (UKPDS), which associated improvements in glycated haemoglobin (HbA1c) with a reduced incidence of long-term diabetes-related complications [2–6]. However, modern clinical practice has moved towards a more holistic approach to diabetes care, with evidence indicating that reductions in blood pressure, serum lipids and body weight are also associated with a reduced risk of diabetes-related complications [7–10]. Guidelines published by the National Institute for Health and Care Excellence (NICE) therefore recommend an HbA1c target of 6.5% (48 mmol/mol) for people with type 2 diabetes managed with lifestyle modifications and one anti-diabetic agent, and a target of 7.0% (53 mmol/mol) for people with type 2 diabetes not adequately controlled by a single glucose-lowering drug, as well as a weight loss target of 5–10% of body weight [11]. Interventions that can provide reductions in blood glucose levels and body weight for people with type 2 diabetes, while providing value for money over the long term, are therefore highly relevant for the National Health Service (NHS) in the UK, where healthcare resources are coming under ever-increasing strain.

Modern therapies for type 2 diabetes, such as glucagon-like peptide-1 (GLP-1) receptor agonists, combine clinically important reductions in blood glucose levels with weight loss and a low risk of hypoglycaemia [12]. Several GLP-1 receptor agonists are approved for use in the UK, including once-weekly injectable treatments semaglutide and dulaglutide. Once-weekly semaglutide 0.5 mg and 1 mg were compared with dulaglutide 0.75 mg and 1.5 mg, respectively, in the SUSTAIN 7 clinical trial, and were associated with greater reductions in HbA1c and body weight in people with type 2 diabetes with inadequate glycaemic control on metformin, with a subsequent long-term cost-effectiveness analysis in the UK demonstrating that these clinical benefits translated to improved life expectancy, quality of life and cost savings over patients’ lifetimes [13, 14].

Since the publication of SUSTAIN 7, further studies have been designed to evaluate higher doses of GLP-1 receptor agonists, to elucidate if further efficacy can be gained without sacrificing safety. The published AWARD-11 trial, also conducted in patients with inadequate glycaemic control on metformin, demonstrated that escalation from dulaglutide 1.5 mg to 3 mg or 4.5 mg provided a similar safety profile while offering clinically relevant, dose-related reductions in HbA1c and body weight [15]. Similarly, the SUSTAIN FORTE trial demonstrated superior reductions in HbA1c and body weight with once-weekly semaglutide 2 mg compared with once-weekly semaglutide 1 mg alongside a similar safety profile [16]. However, as yet, no clinical trial has assessed these newly approved high doses of one GLP-1 receptor agonist vs. any dose of another GLP-1 receptor agonist.

A recent indirect treatment comparison (ITC) has evaluated once-weekly semaglutide 1 mg vs. dulaglutide 3 mg and 4.5 mg, based on the results of SUSTAIN 7 and AWARD-11 [17]. The ITC was performed using the Bucher method, which accounts for cross-trial differences by measuring treatment effects relative to a common comparator arm (in this case, dulaglutide 1.5 mg) and requires only the availability of summary-level data for each trial [18]. This method is considered appropriate if the relative treatment effect can be assumed to be the same across the two trial populations, which was deemed to hold in the populations of SUSTAIN 7 and AWARD-11 (both conducted in people with type 2 diabetes with inadequate glycaemic control on metformin). Results from the ITC indicated that once-weekly semaglutide 1 mg was associated with significantly greater reductions in HbA1c and body weight vs. dulaglutide 3 mg, and similar reductions in HbA1c and significantly greater reductions in body weight vs. dulaglutide 4.5 mg, in people with inadequate glycaemic control on metformin.

Previous long-term analyses have assessed the cost-effectiveness of once-weekly semaglutide in the UK. These include comparisons vs. the lower 0.75 mg and 1.5 mg doses of dulaglutide, once-daily GLP-1 receptor agonist liraglutide 1.2 mg, and sodium-glucose cotransporter-2 (SGLT-2) inhibitor empagliflozin 25 mg [14, 19, 20]. However, to date, no study has evaluated the cost-effectiveness of once-weekly semaglutide 1 mg vs. the higher 3 mg and 4.5 mg doses of dulaglutide, treatments that people with type 2 diabetes in the UK are expected to receive during the course of their disease. The present study was, therefore, designed to answer a pertinent research question for physicians, healthcare payers and people with type 2 diabetes in the UK and to elucidate the relative cost-effectiveness of several highly efficacious GLP-1 receptor agonist therapies.

Based on the results from the ITC, the aim of the present study was to evaluate the long-term cost-effectiveness of once-weekly semaglutide 1 mg vs. dulaglutide 3 mg and 4.5 mg for the treatment of people with type 2 diabetes not achieving glycaemic control on metformin in the UK.

## Methods

### Modelling approach

The IQVIA CORE Diabetes Model (version 9.0) was used to project clinical and cost outcomes over patients’ lifetimes (50 years), in line with guidance on the cost-effectiveness of interventions for diabetes [21]. The structure, assumptions, features and capabilities of the model, as well as two validation studies, have been previously described [22–24]. Relevant model outputs include life expectancy (measured in years), quality-adjusted life expectancy (measured in quality-adjusted life years [QALYs]), direct costs, incremental cost-effectiveness ratios (ICERs), cumulative incidence and time to onset of diabetes-related complications, and cost-effectiveness scatterplots and acceptability curves.

Base case and sensitivity analyses were performed using a first-order Monte Carlo approach, with second-order uncertainty captured in the probabilistic sensitivity analysis (PSA). In line with recommendations from the model proprietors, the UKPDS 68 risk equations were used to predict outcomes in the base case analysis [24]. Future clinical and cost outcomes were discounted at 3.5% *per annum*, in line with guidance published by NICE [25]. Background mortality was captured based on UK-specific life tables published by the World Health Organisation (WHO) [26].

### Baseline cohort characteristics and treatment effects

Baseline cohort characteristics were sourced from the full population in the SUSTAIN 7 clinical trial, as this was used to inform the once-weekly semaglutide arm of the ITC (Table S1) [13, 17]. These cohort characteristics were used in a previously published cost-effectiveness analysis of once-weekly semaglutide in the UK [14]. The mean (standard deviation) age of the cohort was 56 (10.6) years, with a mean duration of diabetes of 7.4 (5.7) years, mean HbA1c of 8.2 (0.9)% [66.1 (10.1) mmol/mol], and mean body mass index (BMI) of 33.5 (6.8) kg/m^2^. Alcohol and tobacco consumption were not collected in the SUSTAIN 7 study and these were therefore assumed to be the same as the general UK population [27, 28].

Treatment effects were taken from the ITC, and comprised changes in HbA1c and BMI (calculated from changes in body weight from the ITC using the mean height from SUSTAIN 7; Table S2) [17]. Once-weekly semaglutide was associated with mean changes in HbA1c and BMI of − 1.78 (standard error 0.06)% and − 2.33 (0.10) kg/m^2^, respectively, while dulaglutide 3 mg was associated with corresponding changes of − 1.54 (0.10)% and − 1.44 (0.16) kg/m^2^. Dulaglutide 4.5 mg was associated with mean changes in HbA1c and BMI of − 1.71 (0.10)% and − 1.67 (0.16) kg/m^2^, respectively. Once-weekly semaglutide 1 mg was associated with statistically significant reductions in HbA1c and BMI vs. dulaglutide 3 mg, and in BMI vs. dulaglutide 4.5 mg. Changes in all other physiological parameters and adverse event rates included in the IQVIA CORE Diabetes Model, including blood pressure, serum lipids and hypoglycaemic event rates, were set to zero in all treatment arms to ensure that assumptions in these parameters did not drive cost-effectiveness outcomes.

### Treatment switching and long-term parameter progression

In the base case analysis, a simple treatment algorithm was employed to evaluate cost-effectiveness, aiming to limit the impact of modelling assumptions on the overall conclusions of the analysis. Modelled patients were assumed to receive once-weekly semaglutide or dulaglutide for 3 years, in line with previously published analyses of GLP-1 receptor agonists and data from general practice in Europe, which reported a mean duration of treatment with GLP-1 receptor agonists of 29.35 months (rounded to 3 years, as treatment switching in the IQVIA CORE Diabetes Model can only occur at the end of an annual cycle) [14, 29–31]. After 3 years, treatment with once-weekly semaglutide and dulaglutide was discontinued and patients were assumed to intensify to basal insulin therapy with insulin Abasaglar® (the most common biosimilar insulin glargine in the UK). Patients remained on basal insulin for the remainder of their lifetimes. Alternative approaches to treatment switching were explored in sensitivity analyses, while different treatment algorithms were explored in pathway scenario analyses.

After the application of changes in HbA1c and BMI in the first year of the analysis, values in each arm were assumed to remain constant while patients received semaglutide or dulaglutide treatment. On intensification to basal insulin, differences were abolished by bringing HbA1c in all arms to 7.0% and returning BMI to baseline. This ensured a balanced cost-effectiveness analysis, with differences in physiological parameters only maintained while there was a difference in the medications received. On treatment intensification, non-severe and severe hypoglycaemic event rates of 408 and 10 events per 100 person-years, respectively, were applied, based on data from the UK Hypoglycaemia Study Group [32].

### Costs and utilities

Costs were accounted from a healthcare payer perspective in the UK and expressed in 2020 pounds sterling (GBP). Captured costs included pharmacy and management costs, based on list prices published on the Monthly Index of Medical Specialities (MIMS) database, and the costs of treating diabetes-related complications, which were based on a published literature review (Table S3) [33, 34]. Costs from the literature review were updated by sourcing up-to-date costs from the most recent publications of annually updated sources, and inflated were necessary to 2020 GBP using the Health and Community Health Services Index published by the Personal Social Services Research Unit (PSSRU) [33, 35–42].

Annual treatment costs were calculated based on resource use from SUSTAIN 7, with 100% of patients receiving concomitant metformin for the duration of the analysis (Table S4). No needle or self-monitoring of blood glucose (SMBG) use was assumed for patients receiving semaglutide or dulaglutide, with one needle and one SMBG test per day assumed to be required once patients intensified to basal insulin.

Utilities were taken from a 2014 review by Beaudet et al. with hypoglycaemia disutilities coming from Evans et al*.* 2013 (published after the literature searches by Beaudet et al*.* had been completed; Table S5) [43, 44]. Beaudet et al*.* reviewed the methods of the identified publications to ensure that they met the criteria of the NICE reference case.

### Key drivers of clinical benefits

A series of analyses were performed to evaluate the key drivers of clinical outcomes. Separate analyses applied the differences in HbA1c and BMI in the once-weekly semaglutide arm in turn, with the other parameter set to the value observed in the respective dulaglutide arm.

### Sensitivity analyses

The extrapolation of clinical results by modelling the long-term consequences is inherently associated with uncertainty. Sensitivity analyses were, therefore, performed to assess the robustness of the base case findings. These included: shortening the time horizon to 35, 20, 10, 5 and 3 years; applying clinical and cost discount rates of 0% and 6% in separate analyses; maintaining the treatment effects on BMI for patient lifetimes; applying the upper and lower 95% confidence interval bounds of the estimated treatment differences in HbA1c and BMI in separate analyses; assuming treatment switching after 5 years; applying the UKPDS progression equation for HbA1c and assuming treatment switching when HbA1c exceeded 7.5%; applying the cost of insulin Semglee® on intensification; increasing and decreasing the direct costs of treating diabetes-related complications by 10%; applying the UKPDS 82 risk equations to predict model outcomes; applying an alternative BMI disutility, giving greater weight to changes in BMI; and PSA [45].

### Pathway scenario analyses

Further, more hypothetical analyses were performed to evaluate the impact of modelling different treatment algorithms on cost-effectiveness outcomes. These aimed to capture more clinically realistic treatment intensification patterns, as per NICE guidelines, but were associated with significant uncertainty regarding changes in risk factors at each intensification step [11]. These analyses included two different pathways:

- Patients were modelled to receive once-weekly semaglutide or dulaglutide for 3 years, at which point a sodium-glucose cotransporter-2 (SGLT-2) inhibitor was added to all treatment arms, and patients continued with both GLP-1 receptor agonist and SGLT-2 inhibitor therapy. After a further 3 years, basal insulin was added to all treatment arms, and patients again continued GLP-1 receptor agonist and SGLT-2 inhibitor therapy. Following a further 3 years, patients discontinued GLP-1 receptor agonist and SGLT-2 inhibitor therapy and were assumed to receive basal-bolus insulin for the remainder of their lifetimes.
- Patients were modelled to receive once-weekly semaglutide or dulaglutide for 3 years, at which point basal insulin was added to all treatment arms, and patients continued with GLP-1 receptor agonist therapy. After a further 3 years, patients discontinued GLP-1 receptor agonist therapy and were assumed to receive basal-bolus insulin for the remainder of their lifetimes.

In these analyses, following the addition of an SGLT-2 inhibitor, HbA1c and BMI were assumed to remain constant (at the levels observed following application of the initial treatment effects with semaglutide and dulaglutide), based on the rationale that additional treatments would be required to maintain glycaemic control, while on the addition of basal insulin, HbA1c was brought to 7.0% and BMI was returned to baseline. On initiation of bolus insulin, no changes in HbA1c were applied, while an increase in BMI was applied based on the ‘insulin-experienced’ multivariate prediction equations published by Willis et al*.* [46]. Non-severe and severe hypoglycaemic event rates were increased to 1,020 and 70 events per 100 person-years, respectively, based on data from the UK Hypoglycaemia Study Group [32].

### Compliance with ethics guidelines

This article is based on previously conducted studies and does not contain any studies with human participants or animals performed by any of the authors.

## Results

### Base case analysis

**Fig. 1:**
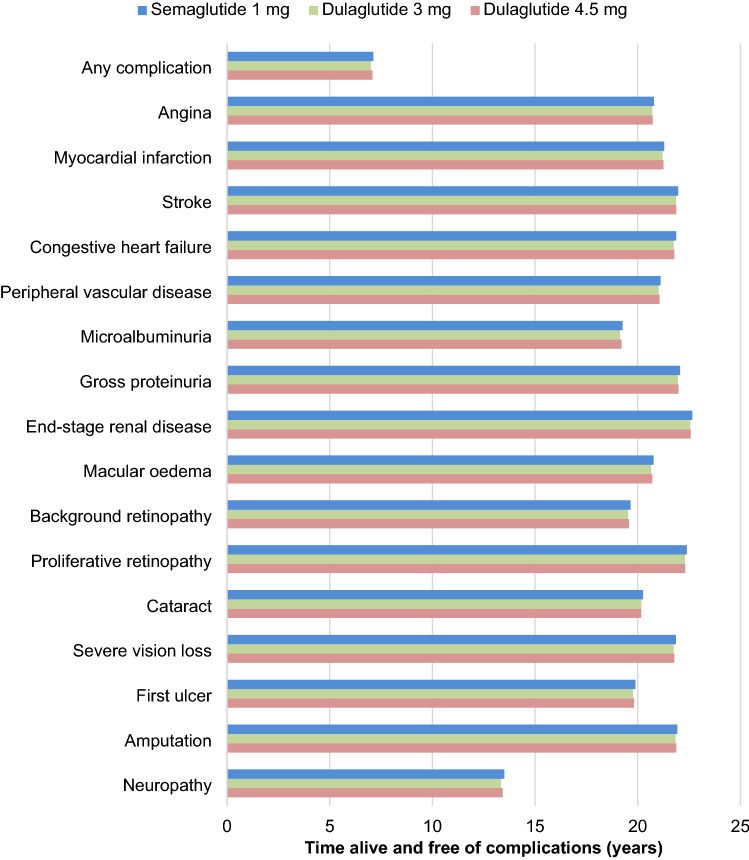
Time to onset of diabetes-related complications over patients’ lifetimes

Long-term projections in patients with inadequate glycaemic control on metformin indicated that once-weekly semaglutide 1 mg was associated with improvements in discounted life expectancy of 0.05 and 0.04 years, and discounted quality-adjusted life expectancy of 0.05 and 0.04 QALYs, vs. dulaglutide 3 mg and 4.5 mg, respectively (Table 1). Clinical benefits with once-weekly semaglutide were a result of a reduced cumulative incidence and delayed time to onset of diabetes-related complications over the long term, with improvements observed in both micro- and macrovascular complications (Fig. 1).

**Table 1:**
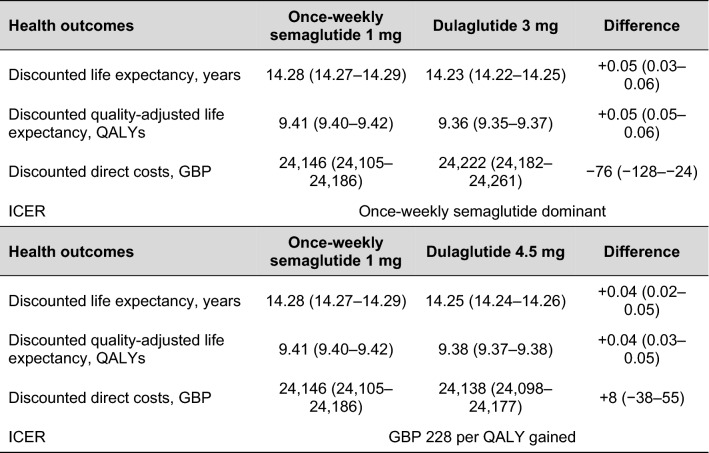
Base case analysis results

*GBP* 2020 pounds sterling, *ICER* incremental cost-effectiveness ratio, *QALY* quality-adjusted life year

**Fig. 2:**
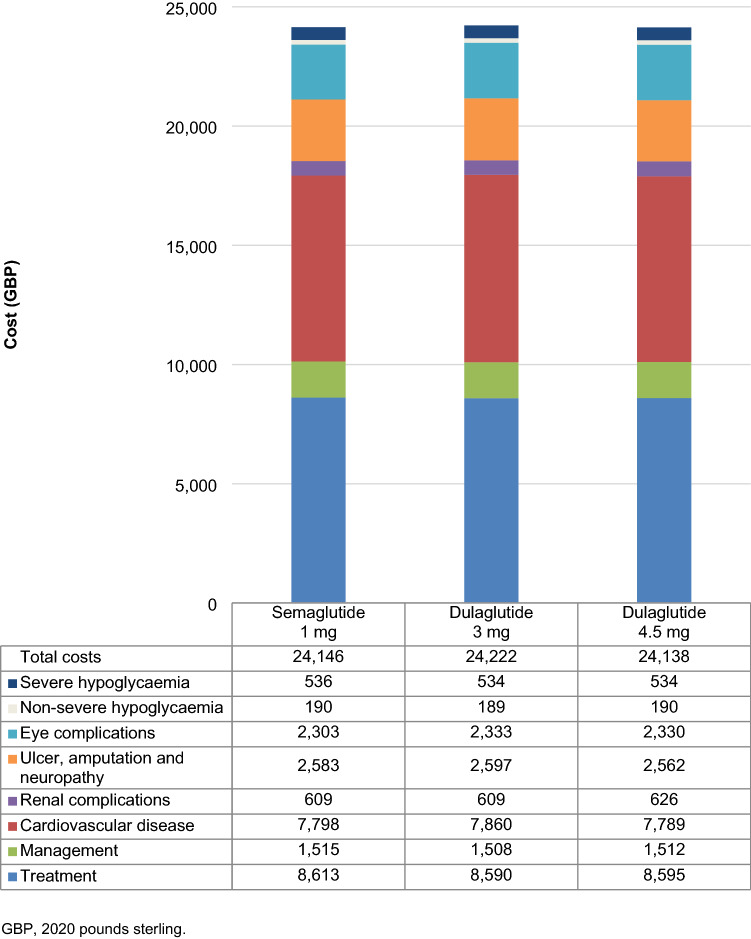
Direct costs over patients’ lifetimes

Direct costs were estimated to be GBP 76 lower with once-weekly semaglutide 1 mg vs. dulaglutide 3 mg, and GBP 8 higher with once-weekly semaglutide 1 mg vs. dulaglutide 4.5 mg (Fig. 2). Once-weekly semaglutide was associated with increased treatment costs in both comparisons despite identical acquisition costs, due to increased survival and further treatment of patients over the long term. In the comparison with dulaglutide 3 mg, increased treatment costs were entirely offset by cost savings from avoidance of diabetes-related complications (most notably cardiovascular complications, with mean cost savings of GBP 62 per patient), while in the comparison with dulaglutide 4.5 mg, increased treatment costs were partially offset by the avoidance of complications (most notably by ophthalmic complications, with mean cost savings of GBP 27 per patient).

Projections of long-term outcomes indicated that both life expectancy and quality-adjusted life expectancy were improved with once-weekly semaglutide 1 mg compared with dulaglutide 3 mg and 4.5 mg, at reduced and increased costs, respectively, from a healthcare payer perspective (Table 1). Overall clinical and cost outcomes were similar, but significantly favoured semaglutide at a 95% confidence level, and it was therefore considered dominant vs. dulaglutide 3 mg and associated with an ICER of GBP 228 per QALY gained vs. dulaglutide 4.5 mg based on modelled mean outcomes for the treatment of type 2 diabetes in the UK.

### Key drivers of clinical benefits

Testing for the key drivers of clinical benefits by applying the differences in HbA1c and BMI in turn showed that reductions in both HbA1c and BMI were equally important in driving improved quality-adjusted life expectancy for once-weekly semaglutide 1 mg vs. dulaglutide 3 mg, with improvements of 0.02 QALYs when each of these differences was applied sequentially. In the comparison with dulaglutide 4.5 mg, greater reductions in BMI were identified as the key driver of benefits, with improvements in quality-adjusted life expectancy of 0.03 QALYs with once-weekly semaglutide 1 mg when only this difference between the treatment arms was applied.

### Sensitivity analyses

Wide-ranging sensitivity analyses showed that the results of the base case analysis were robust to changes in the input parameters and assumptions used (Table 2). In the comparison with dulaglutide 3 mg, once-weekly semaglutide 1 mg remained associated with small but significant clinical benefits and cost savings in all-but-one analysis. When HbA1c was assumed to follow the UKPDS progression equation and treatment switching occurred at a 7.5% HbA1c threshold, once-weekly semaglutide was associated with an ICER of GBP 2,603 per QALY gained. In this analysis, once-weekly semaglutide was associated with increased costs, due to different times of treatment switching in the two arms (with patients receiving once-weekly semaglutide 1 mg for 4 years and dulaglutide 3 mg for 3 years). Patients, therefore, accrued higher treatment costs in the once-weekly semaglutide arm, due to one further year of treatment with GLP-1 receptor agonist therapy.

**Table 2:**
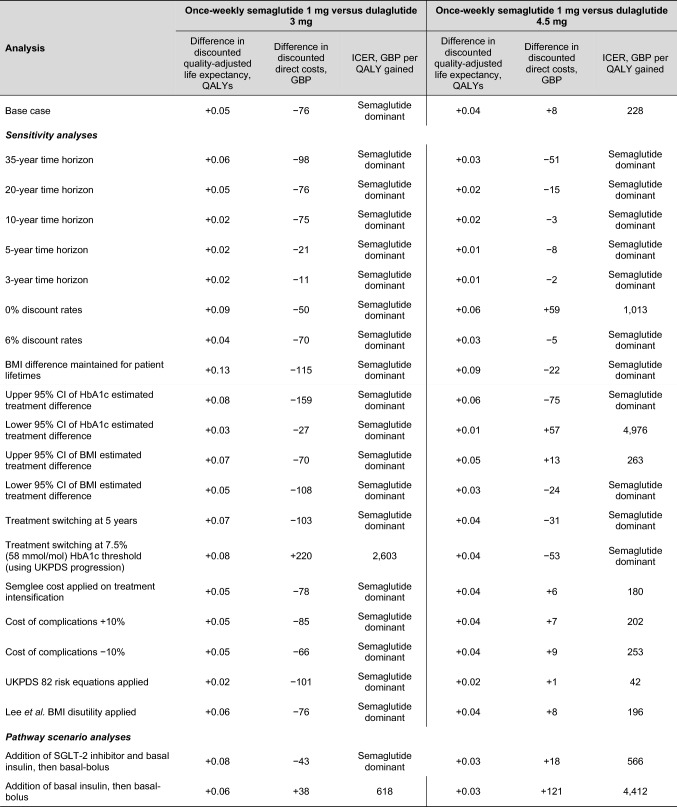
Sensitivity and pathway scenario analyses results

*BMI* body mass index, *CI* confidence interval, *GBP* 2020 pounds sterling, *HbA1c* glycated haemoglobin, *QALY* quality-adjusted life year. Quality-adjusted life expectancy outcomes are rounded to 2 decimal places

In the comparison with dulaglutide 4.5 mg, once-weekly semaglutide 1 mg remained associated with low ICERs in the majority of analyses. The largest deviation in the ICER occurred when applying the lower 95% confidence interval of the estimated treatment difference in HbA1c between the treatment arms, yielding an ICER of GBP 4,976 per QALY gained for once-weekly semaglutide.

**Fig. 3:**
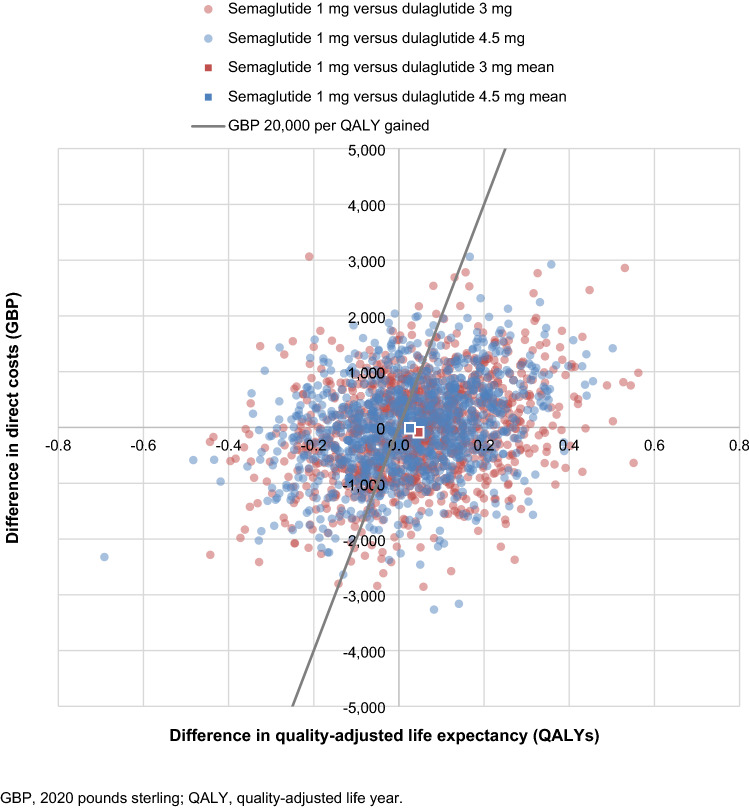
Cost-effectiveness scatterplot from the probabilistic analyses

**Fig. 4:**
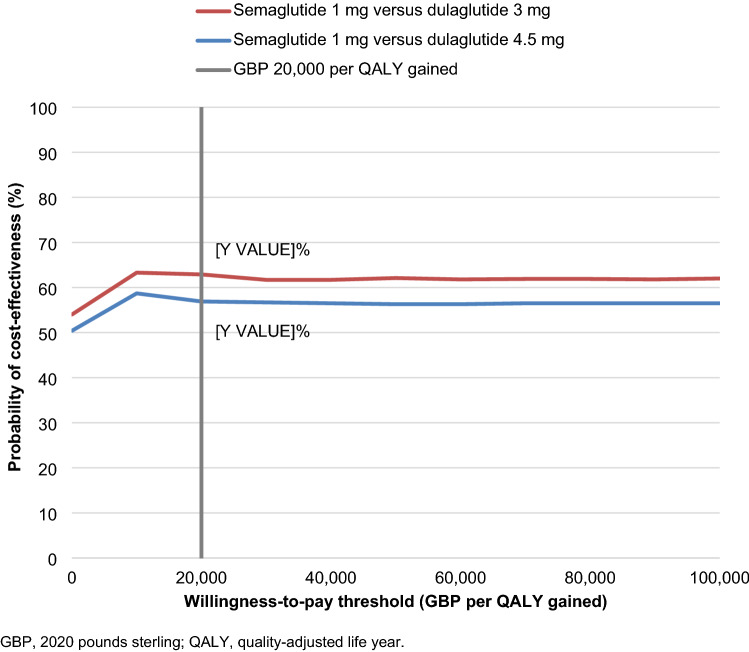
Cost-effectiveness acceptability curve from the probabilistic analyses

PSA showed similar mean results to the base case analysis, but increased measures of variance around the mean outcomes (Fig. 3). Once-weekly semaglutide 1 mg was associated with mean improvements in quality-adjusted life expectancy of 0.05 QALYs vs. dulaglutide 3 mg and 0.03 QALYs vs. dulaglutide 4.5 mg, with direct costs estimated to be GBP 82 and GBP 13 lower, respectively. While these differences were small, they were statistically significant at a 95% confidence level, and once-weekly semaglutide 1 mg was, therefore, considered dominant vs. both doses of dulaglutide in the PSA based on mean outcomes. Based on this analysis, and assuming a willingness-to-pay threshold of GBP 20,000 per QALY gained, the probabilities of once-weekly semaglutide 1 mg being cost-effective vs. dulaglutide 3 mg and 4.5 mg were 62.9% and 56.9%, respectively (Fig. 4).

### Pathway scenario analyses

Hypothetical analyses evaluating different treatment pathways showed that addition of other anti-diabetic agents to GLP-1 receptor agonist therapy did not change the conclusions of the base case analysis (Table 2).

The addition of an SGLT-2 inhibitor followed by the addition of basal insulin and eventual intensification to basal-bolus therapy led to poorer outcomes and increased costs in all treatment arms, due to the further increase in BMI applied on intensification to bolus insulin (following the return of BMI to baseline on initiation of basal insulin) and the increased number of anti-diabetic agents, respectively. Small but statistically significant clinical benefits and cost savings were maintained with once-weekly semaglutide 1 mg vs. dulaglutide 3 mg, and it was associated with an ICER of GBP 556 per QALY gained vs. dulaglutide 4.5 mg.

The addition of basal insulin followed by intensification to basal-bolus therapy led to further reduced life expectancy and quality-adjusted life expectancy in all treatment arms, as the initiation of bolus insulin (and corresponding increase in BMI) was earlier in the analysis. Costs were also increased in comparison with the base case analysis, but reduced in comparison with the addition of an SGLT-2 inhibitor pathway analysis. Once-weekly semaglutide was associated with increased costs in both comparisons, due to increased survival and further treatment of patients over the long term, and was therefore associated with ICERs of GBP 618 and GBP 4,412 per QALY gained vs. dulaglutide 3 mg and 4.5 mg, respectively.

## Discussion

The present analysis has shown that, based on the results of a recent ITC, once-weekly semaglutide 1 mg is likely to represent a cost-effective treatment option vs. dulaglutide 3 mg and 4.5 mg for the treatment of people with type 2 diabetes in the UK. Greater reductions in HbA1c and BMI were shown to result in a reduced incidence of long-term diabetes-related complications, and thereby improved life expectancy and quality of life. These results should inform healthcare payers, physicians and patients in the UK when evaluating potential treatment options for type 2 diabetes.

Once-weekly semaglutide and dulaglutide have been shown to be efficacious in populations with inadequate glycaemic control when treated with metformin in the SUSTAIN 7 and AWARD-11 clinical trials, respectively, and in the ITC [13, 15, 17]. However, the populations evaluated in these clinical studies and in the present analysis did not align with the recommended population for GLP-1 receptor agonists in the current NICE guidelines, which indicate GLP-1 receptor agonists as part of triple therapy for people with type 2 diabetes with a BMI over 35 kg/m^2^, for those whom insulin therapy would have significant occupational implications, or where weight loss would benefit other obesity-related comorbidities [11]. That acknowledged, the joint guidelines released by the American Diabetes Association (ADA) and the European Association for the Study of Diabetes (EASD) recommend GLP-1 receptor agonists as a preferred second-line treatment option in patients with established cardiovascular disease or high cardiovascular risk, and as a potential second-line therapy in populations with a compelling need to minimise hypoglycaemia or weight gain or promote weight loss [12]. Previously published cost-effectiveness analyses have also evaluated once-weekly semaglutide vs. other second-line treatment options, including SGLT-2 inhibitor empagliflozin in the UK, insulin glargine U100 in the Netherlands, and dipeptidyl peptidase-4 (DPP-4) inhibitor sitagliptin in Spain, as well as the lower 0.75 mg and 1.5 mg doses of dulaglutide in people with inadequate glycaemic control on metformin in the UK [14, 19, 30, 47]. In line with results from the present study, once-weekly semaglutide was found to be cost-effective throughout all these comparisons, even with differences in country-specific parameters such as healthcare systems, costs, and mortality. Results from the present study can also add to the current evidence base of cost-effectiveness analyses of once-weekly semaglutide, as described in a systematic literature review published in 2021 [48]. Additionally, sub-group analyses based on baseline HbA1c and BMI have shown that once-weekly semaglutide is consistently more efficacious than other treatment options included in the SUSTAIN trial programme in all sub-groups, rather than just those with a high BMI at baseline [49–51]. Based on this evidence, and the results generated in the present study, once-weekly semaglutide would likely represent an effective treatment while providing value for money if used as second-line therapy in the general population with type 2 diabetes in the UK.

The pathway scenario analyses performed in the present study represent a key strength. While the approach used in the base case analysis, with patients receiving semaglutide or dulaglutide for 3 years before intensification to basal insulin, allowed a fair and direct comparison of two interventions, this approach can be accused of oversimplifying clinical reality, where multiple agents are often required throughout patients’ lifetimes to maintain glycaemic control. These additional pathway analyses thereby compliment the base case analysis by offering further evidence of cost-effectiveness in scenarios that can be considered more clinically relevant. However, the assumptions used to inform these analyses must be considered when interpreting the results. HbA1c and BMI benefits from the ITC were assumed to persist when patients initiated SGLT-2 inhibitor therapy, and HbA1c and BMI were brought to 7.0% and reverted to baseline, respectively, on initiation of basal insulin. These assumptions were used *in lieu* of clinical data informing changes in HbA1c and BMI on initiation of SGLT-2 inhibitor or basal insulin therapy in populations previously receiving metformin and GLP-1 receptor agonist therapy, and this remains the key limitation in modelling more complex treatment algorithms for type 2 diabetes. While the impact of the addition of a GLP-1 receptor agonist to an SGLT-2 inhibitor has been tested in SUSTAIN 9, the impact of adding an SGLT-2 inhibitor in those already receiving a GLP-1 receptor agonist has not been tested [52]. Future studies evaluating changes in physiological parameters on initiation of different treatment classes, stratified by background medication, would therefore provide pertinent information in the diabetes modelling area and allow more clinically relevant treatment algorithms to be accurately assessed.

The present analysis did not include any outcomes from the cardiovascular outcomes trials (CVOTs) of once-weekly semaglutide (SUSTAIN 6) or dulaglutide (REWIND), and this could be seen as a limitation [53, 54]. Indeed, recent guidelines published by the EASD have indicated GLP-1 receptor agonists and SGLT-2 inhibitors as treatments for patients with high cardiovascular risk [12]. However, the incorporation of results from CVOTs into current modelling approaches for type 2 diabetes is a challenge. Based on the currently available data, it is unclear whether changes in known risk factors (such as HbA1c and body weight or BMI) are the primary cause of outcomes observed in CVOTs, or whether there are as-yet-unknown mechanisms of action that wholly or partially influence these outcomes [55, 56]. The present study utilised a model primarily based on UKPDS data, with changes in known biomarkers associated with significant reductions in the incidence of microvascular complications and non-significant reductions in the incidence of macrovascular complications, and the application of further risk reductions in the outcomes evaluated in CVOTs, therefore, risked double counting of benefits. Differences in populations must also be considered, with SUSTAIN 6 and REWIND enrolling patients with a higher cardiovascular risk than those recruited for SUSTAIN 7 and AWARD-11, and outcomes may therefore not be generalisable across studies. Ideally, novel risk equations should be developed incorporating data from CVOTs, and this should be the focus of future research in the area. Calibration of existing models for type 2 diabetes could also offer a stop-gap solution that would allow these data to be utilised in health economic evaluations based on individual CVOTs [56].

A limitation of the analysis, inherent to all long-term modelling studies, was the projection of long-term outcomes from short-term data. However, this is an essential tenet of long-term diabetes modelling, and, given the progressive nature of the disease, arguably represents the best option for informing decision making in absence of long-term clinical trial data. Indeed, projecting outcomes over patients’ lifetimes is recommended in the computer modelling guidance for diabetes interventions [21]. Moreover, every effort was made to minimise clinical doubt, by using a model of diabetes that has been extensively published and validated and by performing a series of wide-ranging sensitivity analyses that were conducted around numerous aspects of the modelling analysis, including the time horizon, treatment switching approaches, and applied reductions in HbA1c and BMI, and these did not change the conclusions of the analysis.

## Conclusions

Once-weekly semaglutide 1 mg is likely to represent a cost-effective treatment option vs. dulaglutide 3 mg and 4.5 mg for the treatment of people with type 2 diabetes with inadequate glycaemic control on metformin from a healthcare payer perspective in the UK.

## Supplementary Information

Below is the link to the electronic supplementary material.

Supplementary file1 (DOCX 225KB)
