# Supplementary material for: The long-term cost-effectiveness of once-weekly semaglutide 1 mg vs. dulaglutide 3 mg and 4.5 mg in the UK
Source: Eur J Health Econ. 2022 Sep 17;24(6):895–907. doi: 10.1007/s10198-022-01514-1 (PMC10290607; doi:10.1007/s10198-022-01514-1)
Supplement: Supplementary file 1 — Supplementary file1 (DOCX 225KB) [file 10198_2022_1514_MOESM1_ESM.docx]

Supplementary material

List of tables

[Table S1 Baseline cohort characteristics applied in the analyses 1](#_Toc107403363)

[Table S2 Treatment effects and adverse event rates applied in the analyses 1](#_Toc107403364)

[Table S3 Costs of treating diabetes-related complications applied in the analyses 2](#_Toc107403365)

[Table S4 Annual treatment costs applied in the analyses 4](#_Toc107403366)

[Table S5 Health-state utilities and event-based disutilities applied in the analyses 4](#_Toc107403367)

Table S1 Baseline cohort characteristics applied in the analyses

| Characteristic | Mean (standard deviation) |
| --- | --- |
| Age, years | 55.7 (10.6) |
| Duration of diabetes, years | 7.4 (5.7)^†^ |
| Male, % | 55.2 |
| HbA1c, % | 8.2 (0.9) |
| Systolic blood pressure, mmHg | 133.0 (14.3) |
| Diastolic blood pressure, mmHg | 80.9 (8.9) |
| Total cholesterol, mg/dL | 181.2 (43.5) |
| HDL cholesterol, mg/dL | 44.8 (10.7) |
| BMI, kg/m^2^ | 33.5 (6.8) |
| Smokers, % | 14.0 |

BMI, body mass index; HbA1c, glycated hemoglobin; HDL, high-density lipoprotein. ^†^ Rounded to 7.0 in the analyses, as the model accepts only integer values for duration of diabetes.

Table S2 Treatment effects and adverse event rates applied in the analyses

| Parameter | Once-weekly semaglutide 1 mg | Dulaglutide 3 mg | Dulaglutide 4.5 mg |
| --- | --- | --- | --- |
| ***Physiological parameters (applied in the first year of the analysis), mean (SE)*** | | | |
| HbA1c, % | −1.8 (0.1) | −1.5 (0.1)* | −1.7 (0.1) |
| Systolic blood pressure, mmHg | 0 (0) | 0 (0) | 0 (0) |
| Diastolic blood pressure, mmHg | 0 (0) | 0 (0) | 0 (0) |
| Total cholesterol, mg/dL | 0 (0) | 0 (0) | 0 (0) |
| HDL cholesterol, mg/dL | 0 (0) | 0 (0) | 0 (0) |
| LDL cholesterol, mg/dL | 0 (0) | 0 (0) | 0 (0) |
| Triglycerides, mg/dL | 0 (0) | 0 (0) | 0 (0) |
| BMI, kg/m^2^ | −2.3 (0.1) | −1.4 (0.2)* | −1.7 (0.2)* |
| ***Hypoglycaemic event rates (applied while patients received treatment)*** | | |  |
| Non-severe hypoglycaemic event rate, events per 100 patient years | 0.00 | 0.00 | 0.00 |
| Severe hypoglycaemic event rate, events per 100 patient years | 0.00 | 0.00 | 0.00 |
| Proportion of non-severe hypoglycaemic events that are nocturnal | 0.00 | 0.00 | 0.00 |
| Proportion of severe hypoglycaemic events that are nocturnal | 0.00 | 0.00 | 0.00 |

BMI, body mass index; HbA1c, glycated hemoglobin; HDL, high-density lipoprotein; SE, standard error.

*Statistically significant difference versus semaglutide 1 mg at a 95% confidence level.

Table S3 Costs of treating diabetes-related complications applied in the analyses

| Complication | Cost, GBP | Reference |
| --- | --- | --- |
| Myocardial infarction, year of event | 8,258 | ^^[[1]](#endnote-1)^^ |
| Myocardial infarction, years 2+ | 2,053 | 1 |
| Angina, year of onset | 2,438 | ^^[[2]](#endnote-2)^^ |
| Angina, years 2+ | 408 | 2 |
| Congestive heart failure, year of onset | 4,690 | 1 |
| Congestive heart failure, years 2+ | 2,751 | 1 |
| Stroke, year of event | 8,881 | 1 |
| Stroke, years 2+ | 2,116 | 1 |
| Stroke, death within 30 days | 4,447 | 1 |
| Peripheral vascular disease, onset | 2,766 | ^^[[3]](#endnote-3)^^ |
| Peripheral vascular disease, years 2+ | 2,766 | 3 |
| Hemodialysis, onset | 21,198 | 3 |
| Hemodialysis, years 2+ | 21,198 | 3 |
| Peritoneal dialysis, onset | 21,218 | 3 |
| Peritoneal dialysis, years 2+ | 21,218 | 3 |
| Kidney transplant, first year | 28,250 | ^^[[4]](#endnote-4)^^ |
| Kidney transplant, years 2+ | 1,318 | 4 |
| Non-severe hypoglycemia | 4 | ^^[[5]](#endnote-5)^^ |
| Severe hypoglycemia | 449 | ^^[[6]](#endnote-6)^^ |
| Laser treatment | 99 | 3 |
| Cataract operation | 787 | 1 |
| Cataract operation, years 2+ | 758 | 1 |
| Blindness, first year | 3,537 | 1 |
| Blindness, years 2+ | 1,340 | 1 |
| Neuropathy, year of onset | 31 | ^[[7]](#endnote-7)^ |
| Neuropathy, years 2+ | 31 | 7 |
| Amputation, procedure | 13,772 | 1 |
| Amputation, prosthesis | 3,827 | 1 |
| Gangrene treatment | 3,745 | ^^[[8]](#endnote-8)^^ |
| Infected foot ulcer | 2,147 | 8 |
| Uninfected foot ulcer | 2,105 | 8 |
| Cost after healed ulcer | 283 | 8 |
| Cost of healed ulcer (history of amputation) | 283 | 8 |

GBP, 2020 pounds sterling.

Table S4 Annual treatment costs applied in the analyses

| Parameter | Cost, GBP | | | |  |
| --- | --- | --- | --- | --- | --- |
|  | Once-weekly semaglutide 1 mg | Dulaglutide 3 mg | Dulaglutide 4.5 mg | Intensification |  |
| ***Study medications*** | | | | | |
| Annual semaglutide cost | 955.52 | ― | ― | ― |  |
| Annual dulaglutide cost | ― | 955.52 | 955.52 | ― |  |
| Annual Abasaglar cost | ― | ― | ― | 343.63 |  |
| ***Concomitant medications*** | | | | | |
| Annual metformin cost | 49.57 | 49.57 | 49.57 | 49.57 |  |
| ***Additional costs*** | | | | | |
| Annual needle costs | 0.00 | 0.00 | 0.00 | 18.23 |  |
| Annual SMBG costs | 0.00 | 0.00 | 0.00 | 75.94 |  |
| **Total annual cost** | **1,005.09** | **1,005.09** | **1,005.09** | **487.37** |  |

GBP, 2020 pounds sterling; SMBG, self-monitoring of blood glucose.

Table S5 Health-state utilities and event-based disutilities applied in the analyses

| Complication | Utility | Reference |
| --- | --- | --- |
| Patient with type 2 diabetes baseline (no complications) | 0.785 | ^[[9]](#endnote-9)^ |
| Myocardial infarction event | −0.055 | 9 |
| Post-myocardial infarction | 0.730 | 9 |
| Angina | 0.695 | 9 |
| Congestive heart failure | 0.677 | 9 |
| Stroke event | −0.164 | 9 |
| Post-stroke | 0.621 | 9 |
| Peripheral vascular disease | 0.724 | ^[[10]](#endnote-10)^ |
| Microalbuminuria | 0.785 | 9 |
| Gross proteinuria | 0.737 | 10 |
| Hemodialysis | 0.621 | ^[[11]](#endnote-11)^ |
| Peritoneal dialysis | 0.581 | 11 |
| Renal transplant | 0.762 | ^[[12]](#endnote-12)^ |
| Background diabetic retinopathy | 0.745 | ^[[13]](#endnote-13)^ |
| Background diabetic retinopathy wrongly treated | 0.745 | 13 |
| Proliferative diabetic retinopathy laser treated | 0.715 | 13 |
| Proliferative diabetic retinopathy no Laser | 0.715 | 13 |
| Macular edema | 0.745 | 13 |
| Severe vision loss | 0.711 | 9 |
| Cataract | 0.769 | ^[[14]](#endnote-14)^ |
| Neuropathy | 0.701 | 10 |
| Healed ulcer | 0.785 | 9 |
| Active ulcer | 0.615 | 10 |
| Amputation event | −0.280 | 9 |
| Post-amputation | 0.505 | 9 |
| Non-severe hypoglycemic event (daytime) | −0.005 | ^[[15]](#endnote-15)^ |
| Non-severe hypoglycemic event (nocturnal) | −0.008 | 15 |
| Severe hypoglycemic event (daytime) | −0.062 | 15 |
| Severe hypoglycemic event (nocturnal) | −0.066 | 15 |
| Each unit of BMI over 25 kg/m^2^ | −0.0061 | 10 |

BMI, body mass index.

References

1. Alva ML, Gray A, Mihaylova B, Leal J, Holman RR. The impact of diabetes-related complications on healthcare costs: new results from the UKPDS (UKPDS 84). Diabetes Med. 2015;32(4):459–66. [↑](#endnote-ref-1)
2. Danese M, Gleeson M, Kutikova L, Griffiths R, Azough A, Khunti K *et al.* Costs of cardiovascular (CV) events in the United Kingdom (UK) using real-world data. Value Health. 2015; 18: A335–A766. [↑](#endnote-ref-2)
3. NHS England. National Tariff Payment System 2020/21. 2020. Available at: https://www.england.nhs.uk/publication/national-tariff-payment-system-documents-annexes-and-supporting-documents/ [Last accessed December 10, 2021]. [↑](#endnote-ref-3)
4. Kent S, Schlackow I, Lozano-Kuhne J, Reith C, Emberson J, Haynes R, Gray A, Cass A, Baigent C, Landray MJ, Herrington W, Mihaylova B, SHARP Collaborative Group. What is the impact of chronic kidney disease stage and cardiovascular disease on the annual cost of hospital care in moderate-to-severe kidney disease? BMC Nephrol. 2015;16:65. [↑](#endnote-ref-4)
5. Chubb B, Tikkanen C. The cost of non-severe hypoglycaemia in Europe. Value Health. 2015;18(7): A611. [↑](#endnote-ref-5)
6. Hammer M, Lammert M, Mejias SM, Kern W, Frier BM. Costs of managing severe hypoglycaemia in three European countries. J Med Econ. 2009;12(4):281–90. [↑](#endnote-ref-6)
7. Monthly Index of Medical Specialities. Available at https://www.mims.co.uk/. [Last accessed December 10, 2021]. [↑](#endnote-ref-7)
8. Ghatnekar O, Willis M, Persson U. Cost-effectiveness of treating deep diabetic foot ulcers with Promogran in four European countries. J Wound Care. 2002;11(2):70–4. [↑](#endnote-ref-8)
9. Clarke P, Gray A, Holman R. Estimating utility values for health states of type 2 diabetic patients using the EQ-5D (UKPDS 62). *Med Decis Making*. 2002; 22(4): 340–9. [↑](#endnote-ref-9)
10. Bagust A, Beale S. Modelling EuroQol health-related utility values for diabetic complications from CODE-2 data. *Health Econ*. 2005; 14(3): 217–30. [↑](#endnote-ref-10)
11. Wasserfallen JB, Halabi G, Saudan P, Perneger T, Feldman HI, Martin PY, Wauters JP. Quality of life on chronic dialysis: comparison between haemodialysis and peritoneal dialysis. *Nephrol Dial Transplant*. 2004; 19(6): 1594–9. [↑](#endnote-ref-11)
12. Kiberd BA, Jindal KK. Screening to prevent renal failure in insulin dependent diabetic patients: an economic evaluation. *BMJ*. 1995; 311(7020): 1595–9. [↑](#endnote-ref-12)
13. Fenwick EK, Xie J, Ratcliffe J, Pesudovs K, Finger RP, Wong TY, Lamoureux EL. The impact of diabetic retinopathy and diabetic macular edema on health-related quality of life in type 1 and type 2 diabetes. *Invest Ophthalmol Vis Sci*. 2012; 53(2): 677–84. [↑](#endnote-ref-13)
14. Lee AJ, Morgan CL, Morrissey M, Wittrup-Jensen KU, Kennedy-Martin T, Currie CJ. Evaluation of the association between the EQ-5D (health-related utility) and body mass index (obesity) in hospital-treated people with Type 1 diabetes, Type 2 diabetes and with no diagnosed diabetes. *Diabet Med*. 2005; 22(11): 1482–6. [↑](#endnote-ref-14)
15. Evans M, Khunti K, Mamdani M, Galbo-Jorgensen CB, Gundgaard J, Bogelund M, Harris S. Health-related quality of life associated with daytime and nocturnal hypoglycaemic events: a time trade-off survey in five countries. Health Qual Life Outcomes. 2013; 11(1): 90. [↑](#endnote-ref-15)
